# Fundamental studies for the proton polarization technique in neutron protein crystallography

**DOI:** 10.1107/S0909049513020815

**Published:** 2013-10-02

**Authors:** Ichiro Tanaka, Katsuhiro Kusaka, Toshiyuki Chatake, Nobuo Niimura

**Affiliations:** a College of Engineering, Ibaraki University, 4-12-1 Naka-Narusawa, Hitachi, Ibaraki 316-8511, Japan; b Frontier Research Center for Applied Atomic Sciences, Ibaraki University, 162-1 Shirakata, Tokai, Ibaraki 319-1106, Japan; c Research Reactor Institute, Kyoto University, 2 Asashironishi, Kumatori, Osaka 590-0494, Japan

**Keywords:** high-sensitivity detection of hydrogen, isotope effect, neutron protein crystallography, proton polarization technique, high-pressure freezing of large biological macromolecules, paramagnetic radical doping

## Abstract

The isotope effect in conventional neutron protein crystallography (NPC) can be eliminated by the proton polarization technique (ppt). Furthermore, the ppt can improve detection sensitivity of hydrogen (relative neutron scattering length of hydrogen) by approximately eight times in comparison with conventional NPC. Several technical difficulties, however, should be overcome in order to perform the ppt. In this paper, two fundamental studies to realise ppt are presented: preliminary trials using high-pressure flash freezing has shown the advantage of making bulk water amorphous without destroying the single crystal; and X-ray diffraction and liquid-chromatography/mass-spectrometry analyses of standard proteins after introducing radical molecules into protein crystals have shown that radical molecules could be distributed non-specifically around proteins, which is essential for better proton polarization.

## Abstract 2

Fundamental trials to realise the proton polarization technique for detecting hydrogen with higher sensitivity in neutron protein crystallography are described.

## 1. Introduction

**Figure 1:**
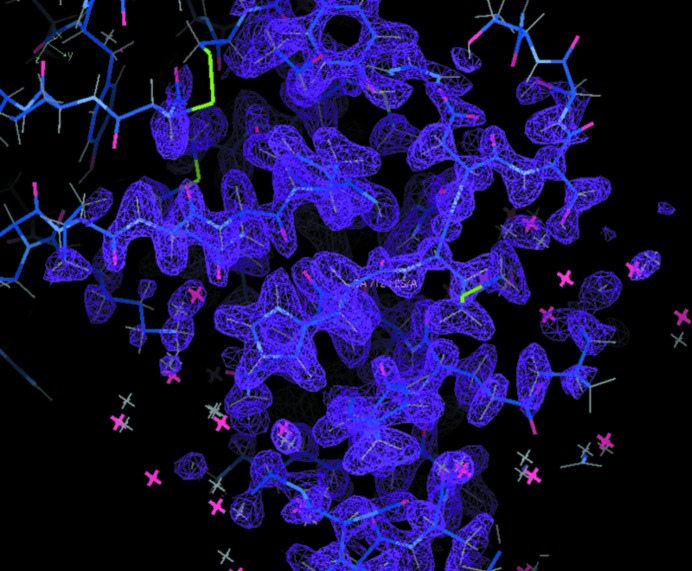
A polarization simulation example of a Fourier map of ribonuclease A. Purple mesh shows the nuclear scattering length at the +2σ level with a model of the protein and waters near a His residue.

Hydrogen, protonation and hydration play important roles in various biological functions at the atomic level. Neutron protein crystallography (NPC) is one of the most powerful techniques for investigating these phenomena concerning hydrogen (Tsyba & Bau, 2002 ▶; Niimura & Bau, 2008 ▶; Tanaka *et al.*, 2010 ▶). However, the hydrogen atom has a large neutron incoherent scattering cross section, which makes the background signal large and the hydrogen atoms sometimes difficult to see when conducting NPC. The current method to reduce this incoherent scattering is deuteration of protein crystals partially by crystalizing proteins or soaking the protein crystals in deuterated buffer made from heavy water. However, there always remains the question of whether three-dimensional structures in deuterated and undeuterated (native) proteins are identical or not, because living cells cannot survive in the heavy water environment for long; in other words, the isotope effect. This effect in conventional NPC can be eliminated by the proton polarization technique (ppt) (Niimura & Podjarny, 2011 ▶). Furthermore, the ppt can improve the relative neutron scattering length (detection sensitivity) of hydrogen by approximately eight times in comparison with the conventional experiment. In Fig. 1 ▶, a polarization simulation example of a Fourier map of ribo­nuclease A [RNaseA; Protein Data Bank (PDB) ID 3a1r] protein is shown in the case of *P* = 0.80 (the number ratio of polarized protons in the protein) with parallel spins, where non-deuterated (native) protein in the light water buffer is assumed, incident neutrons are fully polarized and the resolution limit is 1.70 Å. The visibility of hydrogen becomes better just like the fully deuterated treatment of proteins. This simulation used software programs *PHENIX* (Adams *et al.*, 2010 ▶) and *COOT* (Emsley *et al.*, 2010 ▶).

In order to realise this method, several steps are necessary. At first, almost all of the protons (hydrogen atoms) in all of the protein molecules in a single crystal should be polarized with a proper paramagnetic radical molecule in order to start the polarization, where the ratio of radicals and all hydrogen atoms involved in a protein must be 1:1000 or more. Secondly, the combination of a magnetic field of 3 T, a temperature of less than 1 K and a microwave irradiation is required to polarize the nuclear spins, which is called the dynamic nuclear (proton) polarization (DNP) method. Finally, the incident neutron beam should be polarized parallel and anti-parallel to the direction of the protein protons’ spins as much as possible (∼100%). So far, the technique of producing polarized incident neutrons is well established, and the polarization of protons in protein molecules in solution has been successfully attempted using the DNP method (Stuhrmann *et al.*, 1986 ▶). On the other hand, the polarization of protons in protein molecules in a single crystal has never been attempted. The significant items to be overcome are (i) how to freeze a protein crystal, the volume of which is relatively large, at a higher successful rate, (ii) how to introduce a paramagnetic material uniformly into the crystal sample in order to create the higher proton polarization without destroying the protein structure, (iii) realisation and optimization of a sample polarizer with higher polarization rate, (iv) optimization of incident neutron polarizer instrument, and (v) controlling materials around sample and detector against an intense (∼3 T) magnetic field.

In this paper, two fundamental studies to realise ppt are described: preliminary trials using high-pressure flash freezing showed an advantage of making bulk water amorphous without destroying the single crystal, and X-ray diffraction analysis of standard proteins after introducing radical molecules into protein crystals has shown that radical molecules are distributed non-specifically in proteins.

## 2. Material and methods

### 2.1. Protein single-crystal preparation and high-pressure freezing

Egg-white lysozyme (Seikagaku Corporation, No. 100940) was used as the protein. Crystallization was carried out using the hanging-drop vapor-diffusion technique. The precipitant was a mixture of 18% (*w*/*v*) polyethylene glycol 6000 (PEG6000) and 6% (*w*/*v*) NaCl in sodium acetate buffer (pH 4.5). The protein (25 mg ml^−1^) and the precipitant were dissolved in the buffer. Because the maximum size of the crystal for the high-pressure freezing machine used was less than 2 mm in diameter, each edge of the protein crystal in this cryoexperiment was less than ∼0.5 mm. A high-pressure freezing instrument was used at Japan Aerospace Exploration Agency (JAXA) in Tsukuba, Japan, developed from a prototype by the Cornell University group (Kim *et al.*, 2005 ▶), except that in this case a pressure of 160 MPa was applied. Most samples were pressurized just after scooping the crystal using, for example, cryoloops (Hampton Research), and some, which did not have PEG6000 in the precipitant, were treated by soaking with cryoprotectant such as ethylene glycol 30% (*w*/*v*) and glycerol 20% (*w*/*v*) before cooling. After placing the crystal on a pin in heavy-wall stainless-steel high-pressure tubing, the tubing was fixed to the end of the high-pressure machine, and the lower side of it was immersed in 77 K liquid nitrogen. After the pressure was increased to 160 MPa, ∼3 min, the stopping magnet was released so that the sample pin dropped naturally to the bottom of the liquid-nitrogen-cooled tubing, on a sub-second timescale. Several X-ray diffraction images were recorded using *DIP-2000* (Bruker AXS) under a cryostream of N_2_ at 100 K after removing the sample pin from the tubing in liquid nitrogen and setting it on a magnet pin for a goniometer head of the diffractometer.

### 2.2. Protein single-crystal preparation and paramagnetic radical doping

Lysozyme from chicken egg-white was purchased from Sigma-Aldrich (L6876), ribonuclease A (RNaseA) from Sigma-Aldrich (R5500) and a paramagnetic radical, TEMPO (2,2,6,6-tetramethyl-1-piperidinyloxy free radical), from Sigma-Aldrich (No. 426369). Lysozyme and RNaseA were crystallized using the vapor-diffusion technique. A 20 µl aliquot of 40 mg ml^−1^ lysozyme, 0.5 *M* NaCl and 30 m*M* TEMPO in 0.05 *M* sodium acetate buffer (pH 4.7) was equilibrated to 1.0 *M* NaCl in 0.1 *M* sodium acetate buffer. An aliquot of 60 mg ml^−1^ RNaseA, 10% saturated NaCl, and 25% saturated ammonium sulfate (AS) and 15 m*M* TEMPO in sodium acetate buffer (pH 5.7) was equilibrated to twice the concentrations of NaCl, AS and buffer. Large-scale crystallization of lysozyme was also carried out in order to obtain enough crystals for liquid-chromatography/mass-spectrometry (LC/MS) measurement. A 160 µl solution containing 20 mg ml^−1^ lysozyme, 25 m*M* TEMPO and 1.0 *M* NaCl in 0.050 *M* sodium acetate buffer (pH 4.7) was prepared in a 500 µl microtube. Crystallizations were carried out at room temperature, and protein crystals were grown for one or two weeks. The sample for LC/MS was prepared to solute about 30 crystals in 200 µL pure water.

X-ray experiments were carried out at beamline BL38B1 of SPring-8. X-ray diffraction data sets of lysozyme and RNaseA were collected at 100 K with the oscillation method. LC/MS measurement was carried out using an LCQ Fleet ion trap mass spectrometer (Thermo Scientific).

## 3. Results and discussion

### 3.1. High-pressure freezing of protein crystal

**Figure 2:**
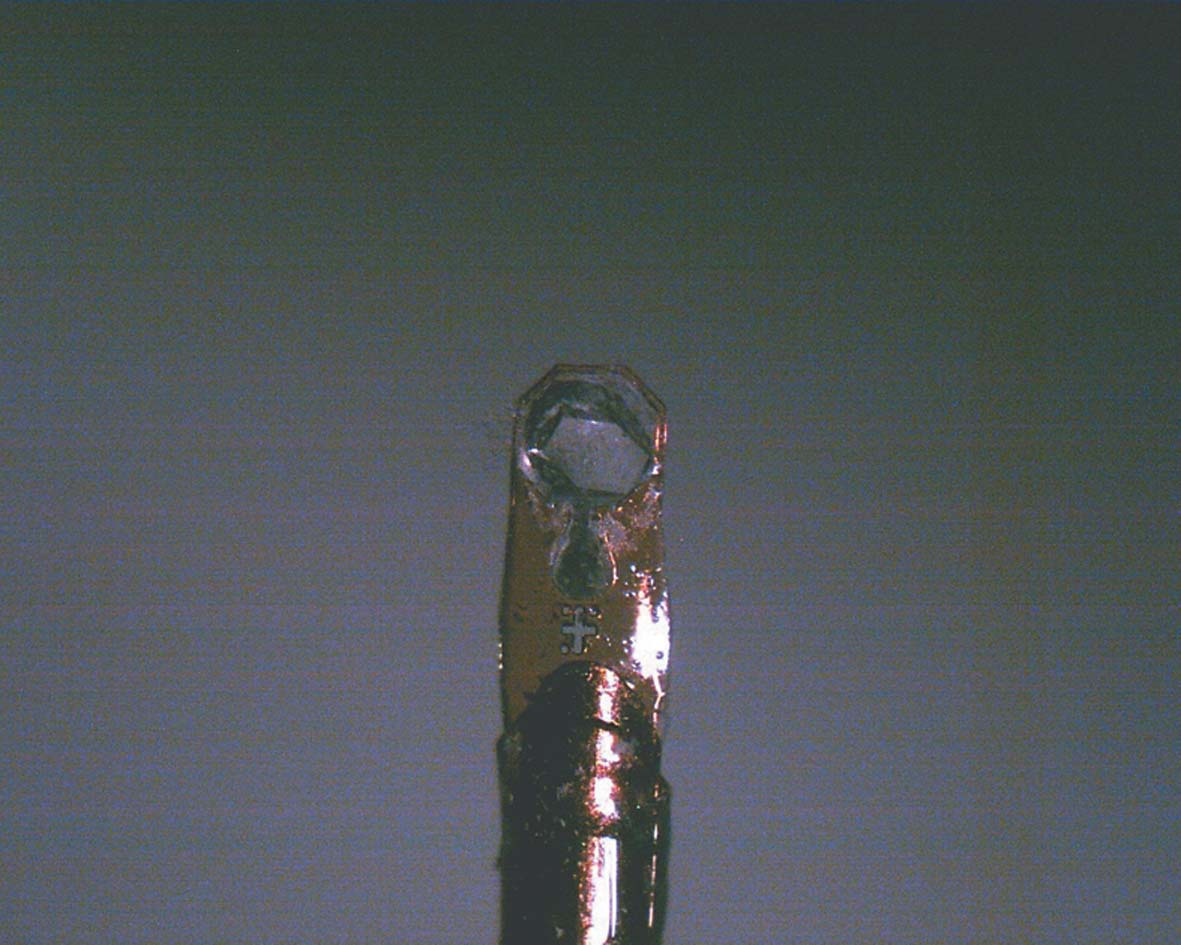
A frozen lysozyme crystal in a cryo-mounting tool.

In order to freeze biological macromolecular crystals without breaking their structures, there are four important requirements: (i) small crystal volume, (ii) rapid cooling, (iii) high pressure around 210 MPa, and (iv) proper doping of the cryoprotectant. Although the first requirement should be overcome because of the inevitable necessity of large crystals in neutron diffraction experiments with weak flux, the latter three are feasible conditions for cooling water molecules in the amorphous state, especially under around 210 MPa pressure where they have maximum viscosity. Lysozyme single crystals grown with the precipitant containing PEG, the dimensions of which were a crystal edge size of around 0.5 mm, were successfully frozen transparently. Crystals without PEG in the precipitant were also frozen in the same way after soaking in cryoprotectants. The reason why they required PEG or some cryoprotectants to freeze successfully may be partly because the used crystals were of relative large size and partly because the applied pressure was 160 MPa, less than 210 MPa. Photographs of a frozen crystal on a cryo-mounting tool in a 100 K cryostream and an oscillation diffraction pattern are shown in Figs. 2 ▶ and 3 ▶, respectively. The crystal seemed clear and the diffraction pattern could be indexed properly up to the same resolution of around 2 Å as the non-frozen one by *DENZO* (Otwinowski & Minor, 1997 ▶). As larger crystals are necessary in an actual NPC experiment, the freezing of relatively large biological crystals requires a more rapid cooling technique, pressures higher than 160 MPa, and more suitable cryoprotectants for a reliable cooling method.

Regarding the stability of the frozen crystals in a cryostat (low temperature around 15 K and vacuum conditions), some reports have shown that biological single crystals diffract well under the above conditions for two or three weeks of neutron measurement (Blakeley *et al.*, 2004 ▶; Myles *et al.*, 2012 ▶). After successfully freezing large crystals in liquid nitrogen, it should be easy to cool them down to less than 1 K because the physical state will not change. It is therefore expected that frozen crystals would be stable at temperatures even lower than 1 K, the DNP conditions.

### 3.2. Paramagnetic radical doping

**Figure 4:**
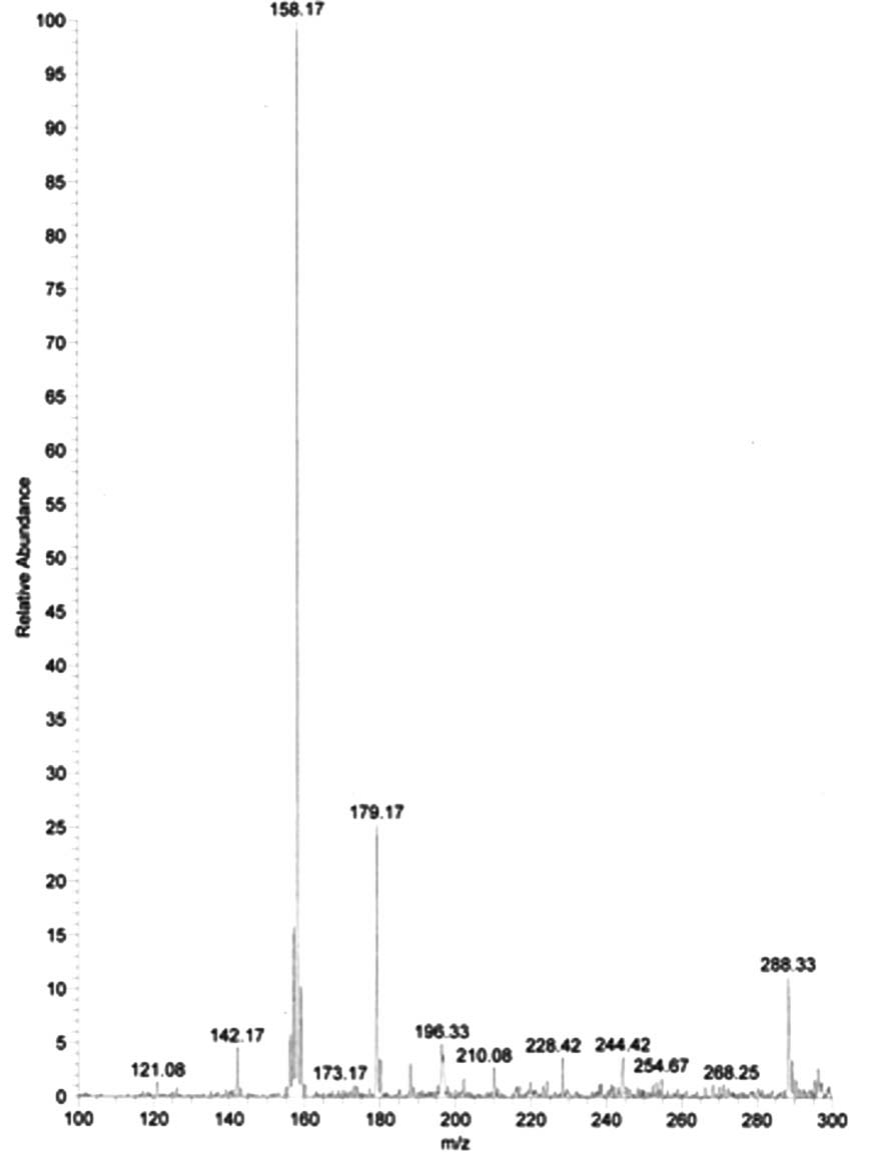
An LC/MS analysis of lysozyme protein after introducing TEMPO molecules.

X-ray diffraction data sets of RNaseA and lysozyme crystals with TEMPO were obtained at 1.8 and 1.45 Å with *R*_merge_ values of 6.4% and 7.0%, respectively. Initial phases of the two crystals were determined by the molecular replacement method, and *R*-factors (*R*_free_) of the refined models were 0.195 (0.216) and 0.192 (0.218), respectively. The results are summarized in Table 1 ▶, suggesting that induction of TEMPO into the two protein crystals did not appear to degrade the crystal quality. The comparison between Fourier maps of crystals with and without TEMPO showed no clear densities corresponding to TEMPO. These results suggested that TEMPO molecules exist in these protein crystals non-specifically (disordered) or never exist. On the other hand, LC/MS analysis on a lysozyme crystal showed that there is a peak corresponding to the molecular weight of TEMPO, 156 Da (Fig. 4 ▶). These facts may indicate that TEMPO molecules are contained in proteins and are distributed non-specifically around the protein. However, there was a possibility of contamination from a very small amount of crystallization solution on the surface of the crystals when picking crystals from the crystallization solution, so a sufficient amount of TEMPO might not be introduced into the crystals. Therefore, for further detailed feasibility studies, electron spin resonance experiments will be required.

## 4. Conclusion

The proton polarization technique is an excellent tool for eliminating the isotope effect and for improving the hydrogen visibility by approximately eight times in comparison with conventional neutron experiments, and it must contribute novel hydrogen information in life and other fields sciences. Although there are several difficulties in the realisation of the ppt in a protein single crystal, two fundamental studies toward realising the ppt were presented: the high-pressure flash-freezing method seems to be promising, and the widely used radical TEMPO may be useful to trigger the polarization of hydrogen atoms in protein because it could be distributed non-specifically.

## Figures and Tables

**Figure 3:**
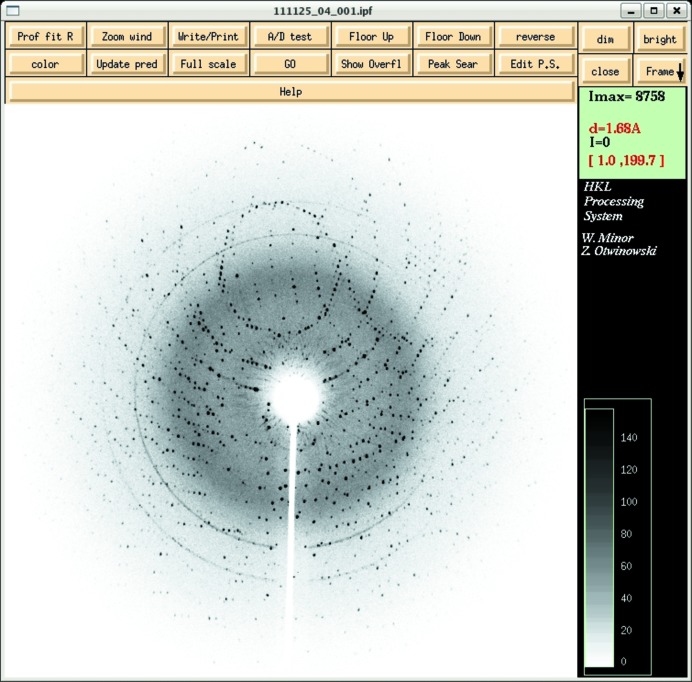
X-ray diffraction image from a frozen lysozyme crystal. The resolution is around 1.8 Å at the edge of the window.

**Table 1:** X-ray diffraction result of two proteins with/without TEMPO

	Lysozyme	RNaseA
Without TEMPO		
Resolution (Å)	1.45	1.45
*R*_merge_ (%)	6.2	5.0

With TEMPO	(30 m*M*)	(15 m*M*)
Resolution (Å)	1.45	1.80
*R*_merge_ (%)	7.0	6.4
